# Effect of Glass Fiber Hybridization on the Durability in Salt-Fog Environment of Pinned Flax Composites

**DOI:** 10.3390/polym13234201

**Published:** 2021-11-30

**Authors:** Vincenzo Fiore, Luigi Calabrese

**Affiliations:** 1 Department of Engineering, University of Palermo, Viale delle Scienze, Edificio 6, 90128 Palermo, Italy; 2 Department of Engineering, University of Messina, Contrada Di Dio (Sant’Agata), 98166 Messina, Italy; lcalabrese@unime.it

**Keywords:** bearing, salt-fog aging, flax, failure modes, hybrid composite laminates

## Abstract

The aim of the present paper is to evaluate the effect of the hybridization with external layers of glass fibers on the durability of flax fiber reinforced composites in severe aging conditions. To this scope, full glass, full flax and hybrid glass–flax pinned laminates were exposed to a salt-fog environment for up to 60 days. Double-lap pinned joint tests were performed to assess the pin-hole joints performances at varying the laminate stacking sequence. In order to better discriminate the relationship between the mechanical behavior and the fracture mechanisms of joints at increasing the aging time, different geometries (i.e., by varying both the hole diameter D and the free edge distance from the center of the hole E) were investigated after 0 (i.e., unaged samples), 30 and 60 days of salt-fog exposition. It was shown that the hybridization positively affects the mechanical performance as well as the stability of pinned composites: i.e., improvements in both strength and durability against the salt-fog environment were evidenced. Indeed, the hybrid laminate exhibited a reduction in the bearing strength of about 20% after 60 days of aging, despite to full flax laminate, for which a total reduction in the bearing strength of 29% was observed. Finally, a simplified joint failure map was assessed, which clusters the main failure mechanisms observed for pinned composites at varying aging conditions, thus assisting the joining design of flax–glass hybrid laminates.

## 1. Introduction

In recent years, the development of sustainable and eco-friendly high-performance materials has been addressed thanks to a growing ecological responsiveness. Hybrid composite materials constituted by polymeric matrices reinforced with both natural and synthetic fibers represent a potentially effective option [1,2]. Tailoring an adequate design of the constituents, a cost-effective, sustainable hybrid composite laminate applicable in structural contexts, could be acquired. 

In this concern, the hybridization of natural fiber reinforced polymers (NFRPs) with glass or carbon one can offer synergistically the benefits of each reinforcement in terms of biodegradability, low weight, high fatigue resistance and low cost for flax fibers [3], good mechanical performance and durability for the glass [4,5] and basalt fibers [6], fatigue properties and long-term durability for the carbon fibers [7].

However, several factors such as fiber content and distribution, and interfacial fiber-matrix adhesion play a role in the performance stability of hybrid structures [8]. Consequently, the design constraints linked to the real operating conditions must be adequately defined in the joining of similar or dissimilar materials. The choice both of the type of fibers and the related stacking sequence contributes significantly not only to the mechanical stability and the failure mode but it has also a key role in the durability of the components in real operating conditions [9,10]. In this regard, several efforts were addressed in the literature to improve the knowledge of the long-term durability of hybrid composites exposed to several critical environmental conditions, e.g., temperature [11], humidity [12] or dynamic loading state [13]. 

Depending on the structure complexity, the component joining is essential to obtain effective structural elements with high mechanical performances and stability [14,15]. In this context, several research activities assessed the mechanical behavior of natural [16], synthetic [17] or hybrid [18] composite pinned joints. However, despite several works in the literature dealing with this research field, few studies have focused on the durability issues in severe environmental conditions of mechanical hybrid composite joints. 

It is well-known that natural fiber-reinforced composites greatly suffer in humid or wet environments [19,20,21,22]. Under these conditions, typical of several application fields (e.g., building, aerospace, nautical, automotive and so on), some structural design issues can emerge due to the difficult identification of the real strength limit of aged materials [23]. To overcome this issue, also avoiding unexpected and premature failures of the joint, a possible solution can be represented by the hybridization of flax fibers by using them together with stronger and more corrosion resistance fibers [24,25]. In this context, it was proved that the use of synthetic fibers, such as glass, as a reinforcement of pinned composite laminates allows to better retain their mechanical performances when exposed to hostile environments such as marine [26]. 

NFRPs show lower mechanical performances than synthetic fiber reinforced ones as well as limited durability when exposed to wet or moist environments during their service life. Therefore, synthetic/natural fiber hybridization is required in potential engineering applications with the aim of improving both the mechanical response and the aging stability in severe environmental conditions such as a marine one [27]. It was shown that the addition of glass fibers plays a positive role in the water uptake of jute fiber reinforced composites, thus leading to an effective strength retention of the resulting composites [28]. Analogously, Almansour et al. [29] and Živković et al. [30] investigated Mode I interlaminar fracture toughness and impact properties of flax/basalt hybrid composites, showing that the hybridization has beneficial effects on the durability in a wet environment of natural flax fiber composites. Furthermore, Fiore et al. [31] used basalt layers as outer laminae in order to improve the aging resistance of flax-reinforced composites exposed to saltwater conditions. As predicted, the percentage of absorbed water was higher for the NFRPs. Moreover, 60-days-aged hybrid composites showed higher strength than full flax reinforced composites. 

These issues become significantly important when a design of complex composite structures such as mechanical fastened joints is required. For the latter, the mechanical stability in aggressive environmental conditions is extremely difficult to predict, thus stimulating the research toward experimental approaches aimed to better define the impact of aging on the degradation of hybrid natural/synthetic fiber-pinned structures. 

In such a context, a comparative study intended to assess the mechanical performance and durability among natural flax, synthetic glass and glass–flax fiber reinforced composites could represent a suitable approach in order to improve the knowledge of this class of materials in the joining design. The aim of the paper is to get deeper insights about the effect of hybridization on the mechanical performance and failure mechanism modifications, induced by aging in a marine environment, of pinned composite laminates. To this scope, hybrid glass/flax pinned composites were exposed to a salt-fog spray condition for up to 60 days. Joints with different geometrical configurations (i.e., by varying the pin/hole diameter and free edge distance of the pin) were also investigated, assessing their mechanical stability of joints at increasing the aging time in this severe environment.

## 2. Materials and Methods

### 2.1. Sample Preparation

Three composite panels (flax, glass and hybrid flax–glass reinforced panels), with dimension 35 × 35 cm^2^, were manufactured through vacuum-assisted resin infusion technique by using a two-stage vacuum pump model VE 235 D by Eurovacuum (Reeuwijk, The Netherlands). This manufacturing method is a close mold technique where fabrics are placed dry into the mold inside a vacuum bag. The dry layup contained in the mold is then impregnated by resin, which flows through driven by vacuum pressure [32,33,34].

**Figure 1:**
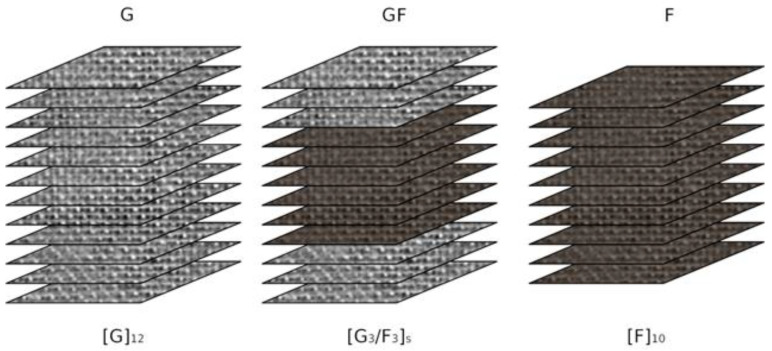
Schematization of stacking sequences of glass, glass/flax and flax laminates.

Each panel was cured for 24 h at 25 °C and then post-cured for further 8 h at 50 °C in the open air. A commercial epoxy resin SX8 EVO (viscosity equal to 500–600 mPas at 25 °C), supplied by Mates Italiana s.r.l., (Segrate, Italy) mixed with its own amine-based hardener (100:30 by weight) was used as matrix. Used as reinforcement were 2 × 2 twill weave woven flax fabrics with nominal areal weight of 318 g/m^2^ (Lineo, Valliquerville, France) and plain weave woven glass fabrics with nominal areal weight of 200 g/m^2^ (Mike Compositi, Milano, Italy). Figure 1 shows the schematization of the stacking sequences of the resulting composites [35,36,37].

All samples were codified by using a prefix composed by a fist letter “G”, ”F” or “GF” indicating the kind of composite (i.e., glass, flax or glass–flax reinforced, respectively) followed by a second letter “A”, “B” or “C” depending on the salt-fog exposition time (i.e., 0, 30 or 60 days, respectively). For instance, the code F-B designates the flax fiber reinforced composite aged for 30 days. 

### 2.2. Salt-Fog Aging 

Glass, flax and glass–flax pinned composites were exposed in a climatic chamber model DCTC 600 (Angelantoni, Massa Martana, Italy) to salt-fog environmental conditions for a whole period of 2 months (i.e., 60 days). According to the ASTM B 117 standard, a 5 wt.% NaCl solution was used to obtain the salt-fog (i.e., pH between 6.5 and 7.2), whereas the temperature inside the chamber was set equal to 35 °C ± 1 °C. 

### 2.3. Pin-Hole Bearing Test

In addition to unaged samples (i.e., reference), pin-hole bearing mechanical tests were carried out on samples aged for 30 and 60 days, respectively. To this aim, a universal testing machine model Z250 (Zwick-Roell, Ulm, Germany) equipped with a 250 kN load cell, was used. The pin-hole bearing resistance of composites was evaluated by performing double-lap pinned joint tests, according to ASTM D5961/D standard (procedure A). All tests were performed at room temperature by setting the displacement rate equal to 0.5 mm/min.

**Figure 2:**
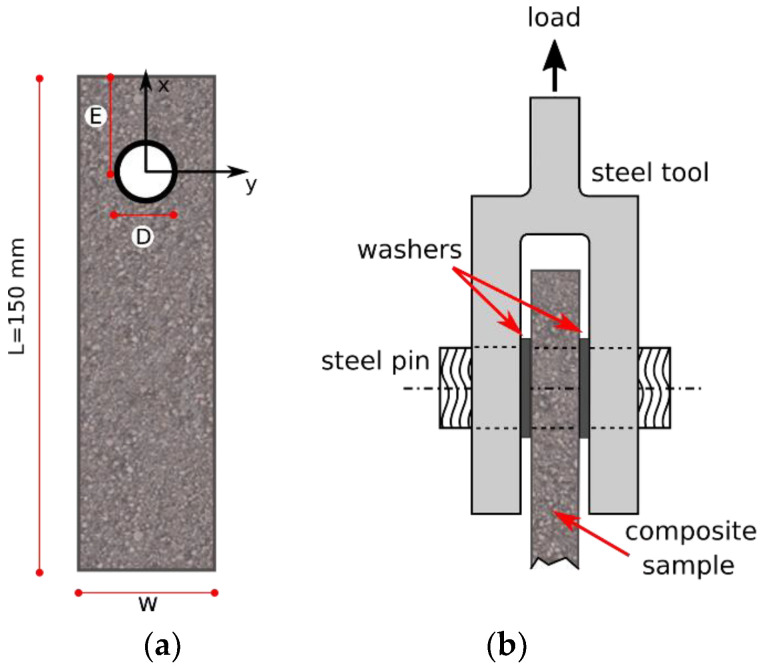
(**a**) Joint geometry and (**b**) scheme of bearing test setup (adapted from [24]).

Figure 2 shows the main geometrical parameters of pinned samples in addition to schematize the pin-hole bearing test setup. For each bearing test, the specimen was bolted by using a steel pin with a specially designed steel plate, in order to reduce its rotation. In particular, the stainless steel pin was positioned in the hole, and then double-lap pinned joint tests were performed by applying a displacement in the direction parallel to the symmetry plane of the composite laminate to hinder undesired moments and rotations. All joints were codified coupling the previous prefix with three numbers related to the joint geometrical configuration. In particular, the three numbers referred to the values in mm of hole diameter (D), free edge distance from the hole (E) and laminate width (W), respectively. In order to have an adequate amount of geometrical condition to investigate, the hole diameter and edge distance were modified within the ranges 4–10 mm and 2–20 mm, respectively. At least 30 joint configurations were characterized for each aging condition, with a total of about 270 specimens.

## 3. Results and Discussion

### 3.1. Stress–Displacement Curves

A preliminary study of the bearing performances of unaged and aged pinned composites can be carried out by assessing the stress–strain curves acquired during the double-lap pinned joint tests at varying the joint configuration as well as the laminate stacking sequence. To this regard, three different geometrical configurations were chosen (i.e., at varying the pin/hole diameter D and the distance between the hole center and the laminate free edge E) for all the investigated composites (glass, flax and hybrid).

The choice of different geometric configurations represents an important aspect to be considered in order to better understand the possible causes of initiation and propagation of the early stage of damage in the pinned joint. By doing so, it can be possible to discriminate the evolution of the main failure mechanisms of composites occurred during the salt-fog exposition.

#### 3.1.1. Small Pin/Hole Diameter D—Small Edge Distance E

**Figure 3:**
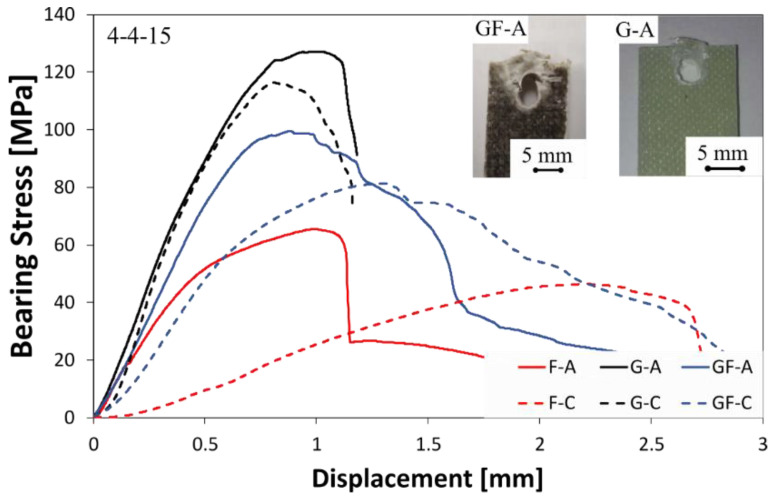
Typical stress–displacement curves of unaged (solid lines) and aged (dotted lines) pinned laminates with D = 4 mm and E = 4 mm.

Figure 3 shows some typical stress–displacement curves of pinned laminates with hole diameter (D) and the laminate free edge (E) both equal to 4 mm, for unaged (solid lines) and aged (dotted lines) samples. This joint configuration was in order to evaluate the mechanical behavior and damage evolution in joints having the pin (or hole) close to the free edge. A clear dependence of the mechanical joint behavior from the stacking sequence can be identified by observing the stress–displacement curves of unaged samples (solid lines). 

Regardless of the stacking sequence, all curves exhibit at low displacement values a linear trend related to the composite joint stiffness. The fiberglass laminate (i.e., G-A) shows the highest slope until a maximum bearing stress is reached. Then, a sudden stress drop takes place due to a premature fracture activation by shear-out. 

Laminates having flax fibers in their stacking sequence also showed a shear-out fracture mode. Nevertheless, the presence of flax fabrics leads to a stiffness decrease, as evidenced by the reduction in the stress–displacement slope for GF-A and F-A samples, in comparison to G-A. This is a quite predicable result since the presence of natural fibers such as flax in the composite stacking sequence obviously exalts a nonlinear behavior of the pinned joint. 

This behavior can be ascribed to the lower elastic modulus and wider elasto-plastic behavior of the flax fiber compared to their synthetic counterpart (i.e., glass) that affects the flexibility of the natural-based composites [31].

The investigated hybrid composite is characterized by coupling stiff glass fibers and flexible flax ones, according to the difference in their failure strains. Thus, it implies that some advantages, compared to flax laminate, are exalted by using a hybrid stacking sequence: (i) an improvement in strength and stiffness is obtained; (ii) the material becomes slightly brittle, although the fracture mechanism is still progressive.

Flax composite (i.e., F-A) shows both the lowest maximum stress and residual stress after the fracture triggering. This behavior can be mainly ascribed to intrinsic properties of natural fibers such as flax, which are less stiff and weaker, as well as more ductile than their synthetic counterpart [38,39].

It is worth noting that the exposition to an aggressive environment such as a salt-fog spray condition noticeably influences the bearing mechanical behavior of composites containing flax fibers in their stacking sequences. Indeed, despite the stress–displacement curves of fiberglass composites not showing clear change due to the salt-fog exposition (i.e., just a slight decrease in the maximum stress value can be noticed by comparing G-A and G-C samples), both GF and F composites experienced evident worsening of their bearing performances. In particular, remarkable decrements in the bearing maximum stress and stiffness, as well as a noteworthy increase in the elongation at break, occurred for flax and glass–flax hybrid-reinforced composites. The higher is the flax fibers content in the composite stacking sequence, the greater are these behavior changes. Although the maximum stretching values of F-C and GF-C batches are quite similar (i.e., about 2.7 mm), noticeable differences can be identified in their stress evolution at increasing the displacement. This is mainly due to the larger softening phenomenon experienced by the F-C sample in comparison to GF-C. 

Although, also after exposition in the salt-fog environment, all of the joints showed a shear-out fracture mode. 

The decrease in performance during aging of the flax-based composite laminates can be mainly ascribed to the scarce durability of natural fibers in humid environments. Flax fibers suffer a relevant water absorption in humid or wet environments, which favors a gradual decrease in the mechanical performances of composites [40]. Furthermore, the hydrophilic nature of natural fibers accelerates the water absorption in addition to promoting its diffusion inside the composite by capillarity at the fiber/matrix interphase [41]. As a consequence, a relevant weakening of composites as well as their reduction in both strength and stiffness occur due to the reduced interfacial shear stress transfer [31].

The water diffusion occurs thanks to hydrophilic areas on the resin matrix and natural fibers that act as preferential pathways [42]. Furthermore, this acts as a driving force for the water flow, by capillarity, into flaws and/or microcracks at the fiber/matrix interface. This contribution is significantly influenced by a low adhesion of the matrix to the fiber, which stimulates the detachment of the interface between them, stimulating the degradation processes in humid environments [43].

Indeed, as clarified in a previous paper [42], the water uptake of flax laminates is relevant after just 10 days of salt-fog exposition (i.e., ~5%) up to reaching a saturation value equal to 12.6% after 60 days of aging. On the other hand, glass laminates exhibited a very limited water uptake at saturation (i.e., 1.1%). Furthermore, glass–flax hybrid laminates experienced intermediate water uptake value at saturation (i.e., 6.9%). 

#### 3.1.2. Small Pin/Hole Diameter D—Large Edge Distance E

**Figure 4:**
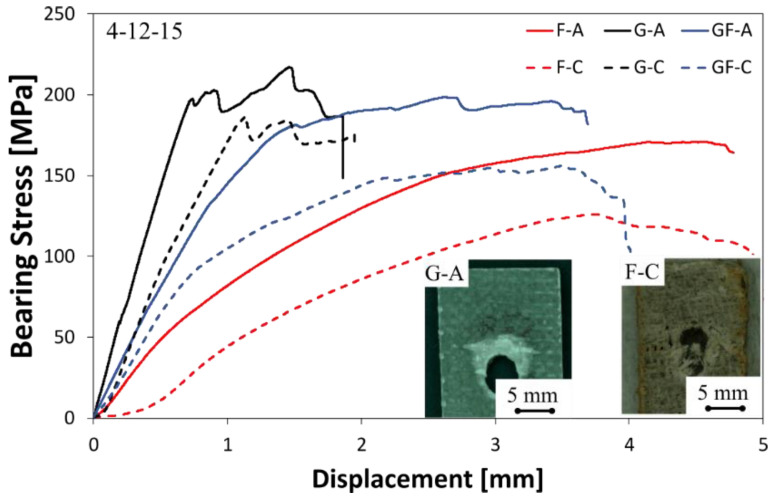
Typical stress–displacement curves of unaged (solid lines) and aged (dotted lines) pinned laminates with D = 4 mm and E = 12 mm.

A joint geometry having a high free edge distance E tends to inhibit a premature shear-out fracture phenomenon (see Section 3.1.1). The choice of this configuration can represent a suitable approach to bring out new fracture mechanisms such as bearing, as well as to evaluate how these mechanisms are influenced by the stacking sequence and aging conditions. In this regard, Figure 4 compares some typical stress–displacement curves of glass, flax and hybrid pinned laminates with D = 4 mm and E = 12 mm. 

For this joint configuration, bearing or net-tension fractures can occur depending on the tensile or compressive limit stress of the laminate [44]. Even in this case, the mechanical behavior of unaged pinned laminates is clearly influenced by the stacking sequence. Indeed, by observing the stress–displacement curves of unaged samples (solid lines), it can be noticed that the fiberglass laminate showed the highest bearing maximum stress and stiffness values as well as the lowest elongation at break among the compared composites. On the other hand, the poorest mechanical response (i.e., in terms of maximum stress and stiffness) was shown by F-A sample, due to the widely known features of flax fibers such as the lower mechanical properties in comparison to their synthetic counterpart (i.e., glass). However, all unaged samples showed mainly a bearing fracture mode for this joint configuration.

All samples exposed for 60 days to salt-fog, exhibited a worsening of their mechanical performances, regardless of their stacking sequence. The applied aging cycle brings a decrease in the stiffness in addition to a slight increase in the elongation at break. This effect is more evident for F batch, for which a relevant nonlinear behavior can be identified. Due to the long exposition to the salt-fog environment, the full flax laminate suffered a reduction in the failure stress equal to 26.2%. 

Conversely, glass fiber reinforced composite showed a quite stable behavior. The joint stiffness, identifiable by the linear initial slope of the stress–displacement curve, suffered a slight reduction. Only a decrease in the failure stress (i.e., −17.6%) can be evidenced, confirming the good mechanical stability of the laminate in this environmental condition. Hybrid aged laminate (GF-C) showed an intermediate trend between G-C and F-C samples (i.e., the variation of their maximum stress compared to unaged laminate is equal to −21.4%).

These findings can be mainly ascribed to the hydrophilic nature of flax fibers [45], which tend to absorb a great amount of water, thus affecting the morphology and, as a consequence, the mechanical response of the resulting composites [46]. Indeed, it is widely known that natural fiber-reinforced composites dramatically suffer the exposition to humid or wet environmental conditions because natural fibers are mainly composed of polysaccharides such as cellulose and hemicellulose. These components present a large amount of strongly polarized hydroxyl groups that easily form hydrogen bonds with water molecules [21,47,48].

Hence, the lower stress/deformation limits of the aged hybrid composite laminate residual than an unaged one promotes the premature compressive failure of the sample around the pin-hole contact region, thus leading to modification of the occurring failure mechanism. Indeed, samples exposed to salt-fog for 60 days failed through a mixed mode between net-tension and bearing failure mode, meaning that a transition from full bearing mode to mixed bearing/net-tension failure mechanism occurs when increasing the aging time.

Further considerations can be argued comparing the occurred fracture mode when varying the stacking sequence and aging conditions. Figure 4 reports the fracture image of G-A sample. All the other unaged samples showed a similar fracture mode.

In particular, this photo clearly evidences that the unaged sample fails on the bearing mode, due to a large compression collapse, probably by the kinking mode [49] of the composite laminate on the pin/hole surface contact area [37]. The local collapse of this reinforced area triggers delamination and matrix cracking, extending the failure zone [50]. During the bearing collapse of the pinned joint, different competing damage mechanisms (i.e., fiber kink bands, delamination, interfacial and shear cracking) occur simultaneously, inducing a premature mechanical instability of the joint [35,51].

As previously assessed, the exposition to a salt-fog environment for 60 days induces a relevant change in the failure mechanism. Both pinned laminates having natural fibers in their stacking sequences (i.e., F-C and GF-C batches) showed a mixed bearing/net-tension fracture mode. As reference, by observing the fracture image of the F-C sample in Figure 4, it is possible to notice that the collapse by bearing in the pin-hole contact area, with a large delaminated zone, is still evident. Nevertheless, unlike unaged samples, kink bands toward the free edges of the sample are not manifest, thus suggesting that other premature failure mechanisms compete in the damage propagation phenomenon. In particular, the matrix softening, due to the epoxy resin aging in the humid environment, hinders brittle fracture events (such as kink bands) as well as triggering local interlaminar or fiber-matrix interfacial fracture mechanisms [52]. This affects the maximum strength of the GF-C and F-C specimens. 

As reported in our previous paper [42], it was found through DMTA analysis that the performance degradation can be ascribed both to the hydrophilic nature of flax fibers (thus influencing both the surface wettability and its water uptake) and to the weak adhesion between flax fibers and the epoxy resin that favor the triggering and propagation of aging phenomena. The flax fabric-based laminate (i.e., GF-C and F-C batches) exhibited large delamination and debonding areas, that synergistically speed up the water diffusion between the laminae and fiber/matrix interfaces [42].

In particular, due to long exposure times in a salt-fog environment, the fracture mechanism observed for GF-C and F-C batches is a mixed bearing and net-tension (i.e., the latter dominating over the former—see image in Figure 4 referred to as reference to F-C). The contribution of the catastrophic net-tension mechanism favors the reduction in the displacement at failure, which does not undergo a significant increase due to aging.

#### 3.1.3. Large Pin/Hole Diameter D—Large Edge Distance E

**Figure 5:**
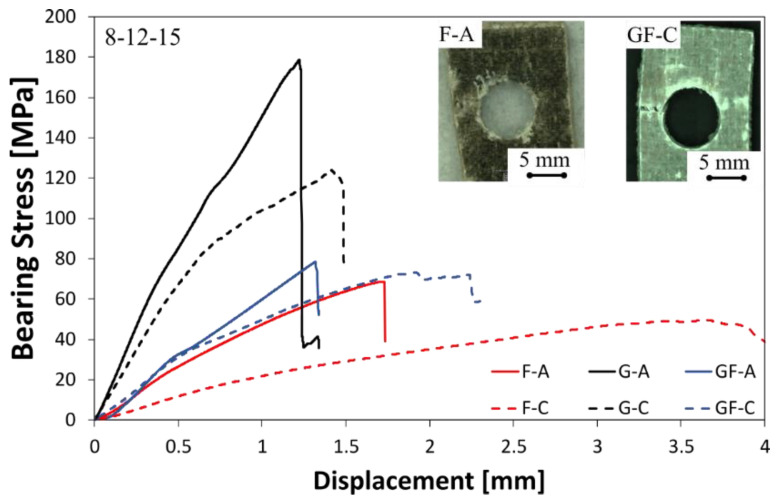
Typical stress–displacement curves of unaged (solid lines) and aged (dotted lines) pinned laminates with D = 8 mm and E = 12 mm.

Figure 5 shows typical stress–displacement curves for pinned laminates with D = 8 mm and E = 12 mm when varying the stacking sequence for unaged and aged samples. The increase in the pin/hole diameter D allows completion of the preliminary investigation of geometrical variables investigated in this campaign, thus providing a preliminary overview of the main damage modes for these classes of materials. The choice of a large hole diameter leads to a significant reduction in the cross-section in the pinned area. Consequently, relevant tensile stresses along the load direction on the pinned transverse plane take place.

As predicted, the reduced cross-section favors premature tensile fractures of the laminate. All samples (some representative curves are compared in Figure 5) showed, therefore, fractures by net-tension mode. This mechanism occurred regardless of both the stacking sequence and the exposure time in the salt spray chamber. Further considerations concerning the mechanical behavior of joints can be argued by evaluating their stress–displacement curves. In comparison to the progressive fracture mainly by the bearing mode observed in Figure 4, all samples having D = 8 mm and E = 12 mm exhibit an abrupt collapse in the stress at break at lower values, due to the occurrence of catastrophic fractures by the net-tension mode. Regardless of the aging time, this brittle behavior is all the more evident the higher the glass fiber content in the laminate. Moreover, significant reductions in joint stiffness and strength can be noticed for the 60-days-aged samples, which lead to a reduction in the performances variance among all batches. Only batch F shows an evident plasticization, which involves a significant increase in the displacement at break. The limited mechanical stability of flax fibers exposed to the humid environment led to the activation of softening and degradation phenomena for this geometric configuration for which the tensile strength of the fiber plays a key role. The hybridization of the flax composite with external glass woven fabric-reinforced laminae (i.e., batch GF) significantly improves the joints’ performance under these environmental conditions. For this stacking sequence, the processes of plasticization and performance decay are strongly hindered, with beneficial effects on the overall mechanical stability. This behavior can represent a valid support for the mechanical joints design in adverse environmental conditions.

### 3.2. Failure Mechanisms at Varying Aging Conditions

**Figure 6:**
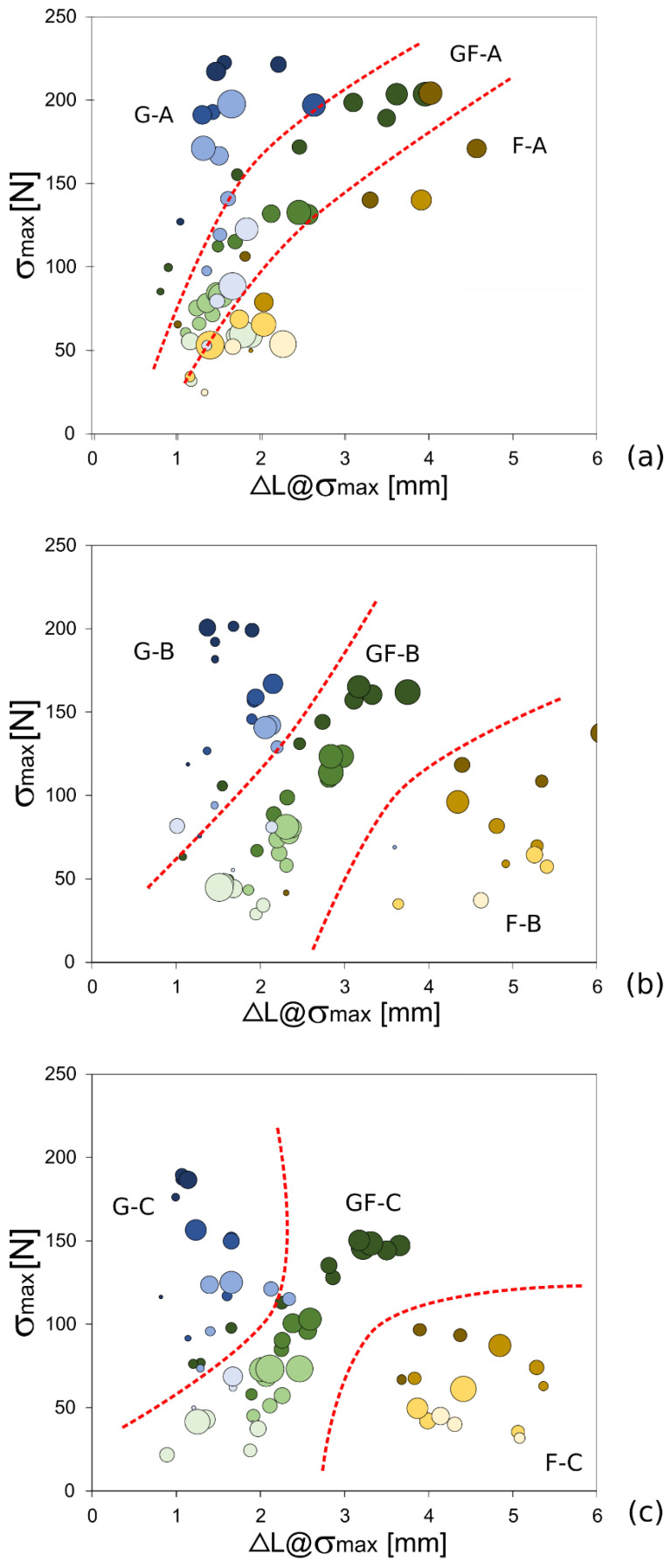
Maximum bearing stress (σ_max_) vs displacement at maximum stress (ΔL@σ_max_) curves for all composite batches at varying the salt-fog exposition time: (**a**) batches A (unaged samples), (**b**) batches B (aging time: 30 days) and (**c**) batches C (aging time: 60 days).

These results indicate that the mechanical performance and stability of the pinned joints are influenced by the stacking sequence and the geometric configuration of the pinned sample as well as the aging conditions. Starting from this, it could be fruitful to extend this investigation in order to improve the knowledge and to better discriminate the relevance of the degradation phenomena on the different composite laminates. To this regard, further details concerning the performance loss evolution of pinned composites can be acquired by evaluating Figure 6. These graphs expose the evolution of the maximum stress (σ_max_) as function of the displacement at the maximum stress (∆L@σ_max_) for all investigated batches at varying the aging exposition time (Figure 6 (a), (b) and (c) for batches A, B and C, respectively). The marker size indicates the free edge distance E (i.e., the larger the marker size, the greater the distance of the pin from the free edge) whereas the marker color brightness indicates the hole/pin diameter size (i.e., the darker the color, the smaller the pin diameter). Finally, dotted red lines were graphically arranged to discriminate the areas of the graphs related to each batch.

Figure 6a shows the results of unaged samples as a function of the stacking sequence. It can be noticed that, regardless of the stacking sequence, gradual increases in both σmax and ΔL@σ_max_ can be noticed when increasing the edge distance (i.e., markers with larger size). At the same time, it can be noticed that samples with a smaller pin diameter (i.e., darker marker color) exhibited higher maximum stress values. This behavior can be justified by considering that for pinned laminates with small D and high E (see in Figure 6a, large size and dark color markers), the main failure mechanism is the bearing mode. As widely known, the latter is a progressive failure mechanism that leads both to higher maximum stress and to displacement at maximum stress values. On the other hand, for small E values (see in Figure 6a small size markers), the laminate suffers premature and catastrophic fractures by shear-out. Concerning this geometry configuration, the composite volume behind the pin, able to bear the stresses acting in the pin/hole contact surface, is very limited. This implies that a sliding of the entire area can be triggered along the load direction, thus leading to shear-our failures of the joint.

A noteworthy descending shift of the markers’ trend can be observed starting from G-A toward GF-A and F-A batches. These latter batches, natural fiber-based composites, show lower maximum load values and larger ΔL@σ_max_ than the G-A batch.

Glass fibers, due to their high strength and noticeable brittleness, can achieve high maximum stresses and low displacement upon failure of the related composites. This implies that the point cloud of the G-A batch is located in the upper left corner of Figure 6a. Conversely, the presence of flax fibers in the composite laminate induces a significant decrease in both composites’ strength and stiffness. This is revealed in the full flax batch (F-A), for which the markers are shifted in the right side of the figure. 

A similar trend can be found in Figure 6b,c referred to samples exposed to salt-fog for 30 and 60 days, respectively. Already after 30 days of aging in the chamber (Figure 6b), the F-B laminate exhibits a significantly lower mechanical behavior compared to the unaged one. The points related to F-B batch are placed at the bottom right of the graph, indicating that the composite material has undergone a significant plasticization phenomenon with a clear reduction in the maximum stress as well as an evident increase in the deformation at maximum stress. 

Furthermore, there is a clear deviation in performance compared to the G-A and GF-A batches (markers of these batches are positioned much higher on the left in Figure 6a) for which an acceptable stability of the mechanical behavior is noticed. Flax fibers, due to their hydrophilic nature, are strongly affected even after a short exposition to humid environments. The absorption of water in flax fibers is significant [46,47,53] and conveys diffusion phenomena, both in the matrix and at the fiber-matrix interface. Thus, the softening and plasticization of the NFRP occur prematurely with a significant decrease in performance.

The presence of glass fibers as reinforcement of the external laminae in the stacking sequence of the hybrid laminate triggers a water shielding effect. Indeed, the GF-B batch clearly shows better mechanical stability at intermediate aging time. Regardless of the joint geometry and related fracture mechanisms, the performances are slightly lower than in the unaged one. This behavior is clearly amplified by analyzing G batches (i.e., full glass fiber composite). By analyzing Figure 6b, it is noteworthy that the hybridization of the flax composite resulted in a valuable increase in the durability, in the salt-fog environment, of pinned composites.

Based on these findings, an important aspect is to evaluate the beneficial effect of the hybridization even at long aging times. Figure 6c shows the data referring to batches C (i.e., exposed for 60 days to salt-fog environment).

Quite interestingly, all batch areas are located slightly at lower stress values. Furthermore, there is no significant increase in ΔL@σ_max_ values compared to batches B. This means that the degradation phenomena activated in the composite laminates, at first leads to a significant increase in the composites flexibility, and then to the stiffness reduction. In other words, the water absorption experienced by flax fibers, which could be considered as the main cause of this phenomenon, takes place at short immersion times [54]. For longer aging times, kinetically slow diffusion phenomena such as water permeation take place through both the matrix bulk and at the fiber/matrix interface. These last phenomena are responsible for the further decay of the mechanical performance of the pinned composite joint. This behavior is mainly evidenced by the batch F-C, which is located in the narrow area at the lower right of Figure 6c.

**Figure 7:**
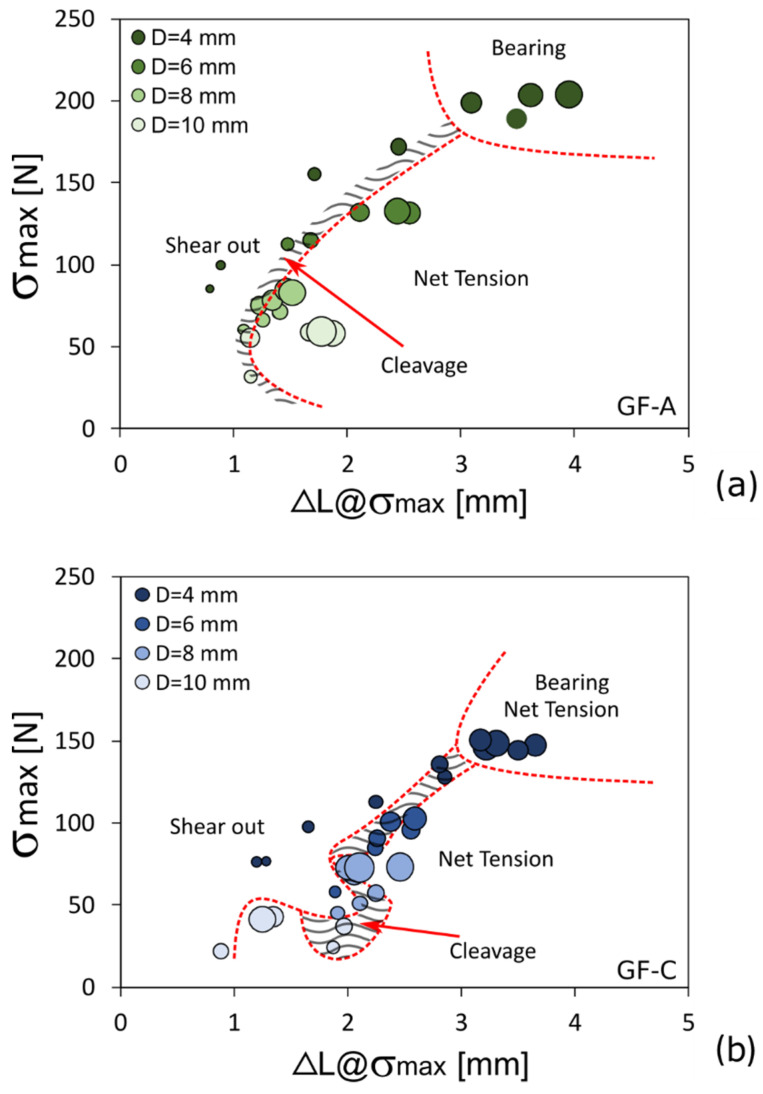
Failure mechanisms evolution in σ_max_ vs ΔL@σ_max_ plot at varying the salt-fog exposition time: (**a**) batch A (unaged samples) and (**b**) batch C (aging time: 60 days) for hybrid laminate, as reference.

The degradation phenomena induced by salt-fog exposition do not only involve a change in the laminate performance but they also affect, depending on the joint geometry, the failure mode [55]. In this regard, Figure 7 shows the experimental data of hybridized composite joints, at varying their geometry, clustered based on the fracture mechanisms that take place. Similarly to the previous figure, the marker size and color brightness indicate the free edge distance E and pin/hole diameter D, respectively. Dotted red lines were graphically added to discriminate better the failure mode transition. 

Analyzing Figure 7a, referring to the GF-A batch, it can be observed that a good discrimination of clusters associated with the main fracture mechanisms is identifiable. In particular, the bearing is characterized by the highest strength and the greatest deformation at the maximum stress. This is attributable to the progressive compressive collapse of the composite that characterizes this fracture mechanism that favors a large displacement at break. Conversely, catastrophic fracture mechanisms such as net-tension and shear-out, can be associated with clusters located at lower ΔL@σ_max_ values. In the transition zone between these two fracture mechanisms, a mixed fracture due to cleavage is detected. A similar topological map could also be found for the other batches.

Figure 7b refers to the GF-C batch, exposed for 60 days to salt-fog environment. Albeit the map topology is preserved, significant differences can be found. A clear shift of the failure mechanism clusters at higher σmax and lower ΔL@σ_max_ values occurs. Furthermore, greater irregularity in the distribution of catastrophic fracture typologies takes place. Net-tension and shear-out exhibit occasionally interconnected clusters. Even the cleavage cluster expands, and it extends in a disordered way in the border area between the two mechanisms. This behavior indicates that aging conditions significantly affect the mechanical stability of the joint and the related fracture mechanisms. This implies that an explicit discrimination is detectable only between progressive (i.e., bearing) and catastrophic (i.e., shear-out and net-tension) mechanisms. The latter, although locally discriminated due to aging, exhibit a partial overlapping in their mechanical behavior, thus leading to a less effective damage clustering. 

**Figure 8:**
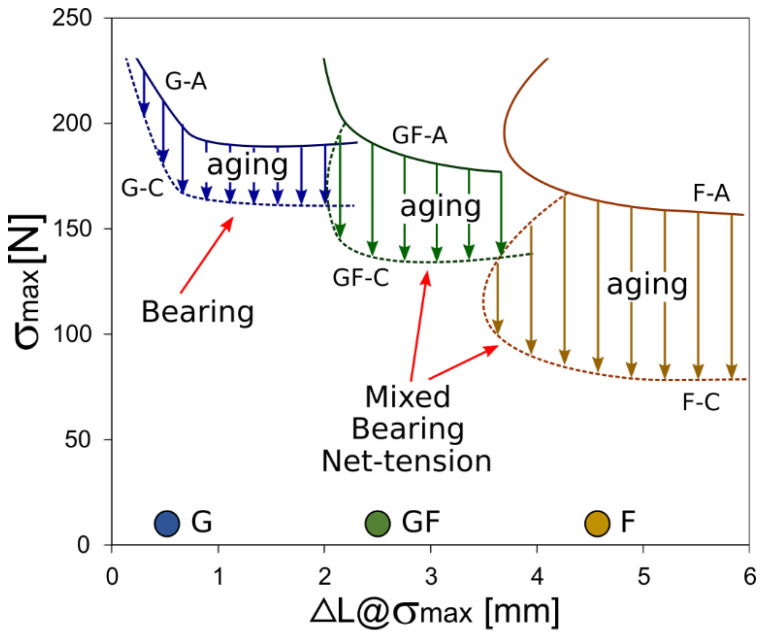
Bearing failure mechanism modification due to aging in σmax vs ΔL@σmax plot at varying stacking sequence.

In order to obtain further insights into the effect of the hybridization on the durability, in a salt-fog environment, of pinned composites, Figure 8 compares the progressive bearing failure modification area due to the aging in the maximum bearing stress (σmax) vs displacement at maximum stress (ΔL@σ_max_) plot. It is important to specify that a transition from bearing to a mixed bearing/net-tension mode occurred for the GF batch. On the other hand, no bearing fractures were observed for the F batch even at long aging times. Even for joints with low D and high E values, the fracture occurred by a mixed mode between bearing and net-tension mechanisms.

For the G batch, the bearing failure transition suffered a limited modification due to aging. Comparing the unaged state (solid blue line) with the aged one (dotted blue line) a slight reduction in performance can be identified (the arrows refer to the reduction induced by aging). The natural flax composite (i.e., F batch) shows an almost different behavior. Due to aging, the bearing area significantly decreased in magnitude, reaching σmax value of about 75 MPa, about 50% lower than the unaged one. It is worth nothing that the observed joint fracture was a mixed bearing and net-tension both in aged and unaged conditions.

The hybridization with glass fibers induced a beneficial effect on the bearing strength and failure mechanism in the flax laminate. The GF batch exhibited, in the aged condition, a bearing fracture mode with acceptable failure stress (above 170 MPa—solid green line). However, after 60 days of exposure in a salt spray condition, the composite underwent a mechanical decay, albeit limited, which led to a mixed bearing net-tension fracture and stress values approximately 20% lower than the unaged one. The evidence of the net-tension fracture can be associated with the reduction in the tensile strength of the flax natural fibers that tend to reduce, especially due to an environmental degradation, the failure strength limit due to this fracture mode [23]. Nevertheless, the hybridization allows preserving, partially, an acceptable mechanical stability of the joint. It is a suitable compromise in performance and durability between the full glass and full flax laminates (G and F batches, respectively).

**Figure 9:**
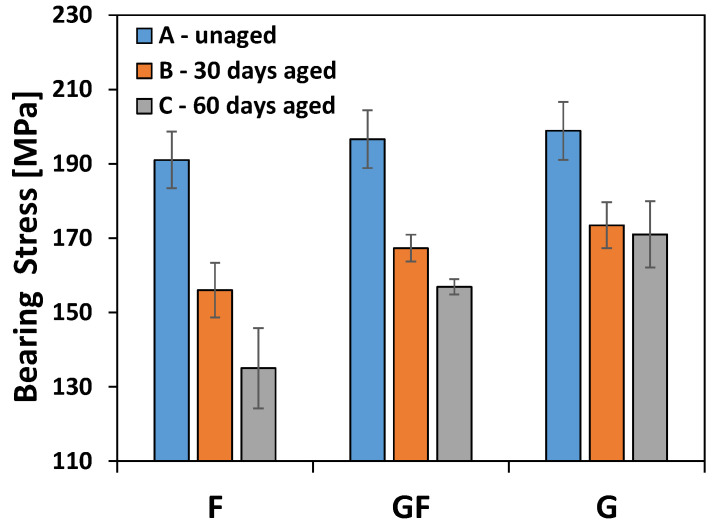
Bearing stress limits experimentally determined for all pinned laminates when varying the salt-fog exposition time.

Figure 9 shows the evolution of the stress limits for bearing fractures when increasing the exposition time to the salt-fog environment (i.e., 0, 30 and 60 aging days for batches A, B and C, respectively) for all pinned laminates. This limit bearing stress was defined as the average maximum strength value considering only joint configurations that failed through bearing mode. In particular, this average value was determined for each batch and for each aging time. 

A relationship between the bearing stress and aging time can be clearly identified. The bearing limit stress of F-B batch (i.e., aged for 30 days in the salt-fog environment) showed a reduction of about 18% compared to the unaged one (F-A batch). Furthermore, no bearing fracture was observed after 60 days of exposition in the salt-fog environment (batch F-C). All samples, in this batch, exhibited only a premature and catastrophic fracture by shear-out or net-tension modes, confirming the detrimental effect of the hostile environment on the mechanical stability and durability of joints. On the other hand, the full glass laminate exhibited a quite stable behavior. A reduction in bearing stress is identifiable of a maximum of about 14% due to 60 days of aging. 

The hybrid unaged laminate (i.e., GF-A batch) is characterized by an intermediate average limit stress at bearing fracture among the investigated laminates. Nevertheless, it is worth noting that the limit bearing stresses of unaged samples are slightly different. Its mechanical performances undergoe a clear decay induced by the degradative phenomena that are triggered during the salt-fog exposition. However, the presence of external glass skins in the stacking sequence allows to acquire an effective shielding action against the diffusion of water. This is confirmed by the non-catastrophic decay of performance as observed for the flax batch. Furthermore, a progressive bearing fracture occurs even at long aging times (i.e., batch C), thus avoiding the premature activation of unwanted catastrophic fractures (i.e., net-tension and shear-out).

The diffusion of water at the fiber-matrix interface plays a key role in triggering and propagating the degradation mechanisms [22]. This induces both the matrix softening and the weakening of the fiber-matrix interface itself [56,57]. It is important to highlight that the shear and bearing resistances of the mechanical joints are mainly influenced by the interfacial adhesion and the matrix stiffness, respectively [58,59]. Nevertheless, the net-tension strength of pinned laminates is significantly affected by the tensile strength of the laminate [60]. Consequently, the aging time has a more marked influence on the bearing strength and fracture mode of natural fiber composite joints. Hence, this implies that the glass/flax fiber hybridization has an important role in the tailoring and designing of the pinned composite joints in order to optimize the material stability and durability in severe environmental conditions. Furthermore, thanks to the beneficial effect of glass fibers in terms of strength and stiffness, a suitable balance of the mechanical performance is achieved, preserving the environmental sustainability of the natural fiber composite laminate. 

## 4. Conclusions

The influence of glass fiber hybridization on the durability of pinned flax composites in a salt-fog environment was evaluated in the present paper. In particular, the effects of the fiber characteristics and the joint geometrical configuration on the maximum strength and failure mechanisms of laminates were assessed at increasing aging time. All samples were exposed to salt-fog spray conditions (i.e., for 0, 30 and 60 days), according to ASTM B 117 standard.

The experimental results evidenced that the hybridization with glass fibers of flax based laminates has a beneficial effect on their durability. Indeed, the hybrid laminate showed a bearing strength reduction of about 20% due to the exposure for 60 days in the salt-fog environment (i.e., GF-C batch versus GF-A). A similar result was found for the full glass laminate, which experienced a reduction in the bearing strength equal to 14% (i.e., G-C batch versus G-A batch). On the other hand, the flax laminate suffers more than the others in the humid aging conditions, showing a noticeable decrease in the bearing strength (i.e., −29%) at the end of the aging campaign. 

Furthermore, a significant change in the fracture pattern was found when increasing the exposition time to salt-fog. In particular, the bearing fracture mechanism was progressively less evident, thus favoring premature and catastrophic failure modes such as shear-out and net-tension, all the more relevant the greater the content of flax fibers in the laminate. In particular, the hybridization preserved the bearing failure mechanism even at long exposition times (i.e., GF-C batch), preventing the triggering of unwanted catastrophic failure modes.

These promising results clearly indicate that the glass/flax hybridization has a key role in the tailoring and designing of pinned composite joints with suitable stability and durability in severe environmental conditions. 

## Data Availability

The data presented in this study are available on request from the corresponding author.
